# Association of Dietary Protein Intake and Grip Strength Among Adults Aged 19+ Years: NHANES 2011–2014 Analysis

**DOI:** 10.3389/fnut.2022.873512

**Published:** 2022-05-13

**Authors:** Matthew A. Pikosky, Christopher J. Cifelli, Sanjiv Agarwal, Victor L. Fulgoni

**Affiliations:** ^1^National Dairy Council, Rosemont, IL, United States; ^2^NutriScience LLC, East Norriton, PA, United States; ^3^Nutrition Impact, LLC, Battle Creek, MI, United States

**Keywords:** animal protein, essential amino acids, muscle strength, National Health and Nutrition Examination Survey, plant protein, protein quality, protein distribution, total protein

## Abstract

**Background:**

Research on the role of protein in the diet has evolved beyond a focus on quantity to include the impact of its quality and distribution across meal times in an effort to optimize dietary protein recommendations.

**Objective:**

To determine the association of dietary protein amount, type, and intake pattern with grip strength in adults.

**Design:**

Data from the National Health and Nutrition Examination Survey (NHANES) 2011–2014 for adults 19 + years (*N* = 9,214) were used with exclusions for pregnant and lactating women. Intakes of dietary total protein (TP), animal protein (AP, including dairy), plant protein (PP), and leucine (Leu) were determined using day 1 24 h dietary recall data after adjusting for the complex sample design of NHANES. Regression analyses were used to assess the association of dietary protein and leucine intake quartiles, and whether consuming > 20 g of dietary protein at one or more meals was related to grip strength with adjustment for age, gender, and ethnicity.

**Results:**

Mean intake of TP among adults aged 19 + years was 83.6 ± 0.5 g/day, and 2/3rd of this was from animal sources (including dairy). Grip strength increased (*p* < 0.05) with increasing quartiles of TP, AP, PP, and leucine among all adults 19 + years (β = 1.340.19, 1.27 ± 0.19, 0.76 ± 0.20, and 1.33 ± 0.23, respectively), 19–50 years (β = 1.14 ± 0.27, 1.06 ± 0.25, 0.77 ± 0.30, and 1.18 ± 0.27, respectively), and 51 + years (β = 0.95 ± 0.26, 1.08 ± 0.27, and 1.05 ± 0.27, respectively, for TP, AP, and Leu); however, the increase was more pronounced for AP than PP. Grip strength also increased (*p* < 0.05) with increasing the number of meal occasions containing > 20 g of dietary protein (β = 1.50 ± 0.20, 1.41 ± 0.25, and 0.91 ± 0.37 for 19+, 19–50, and 51 + years, respectively), and significant increases were detected for two meals compared to zero meals.

**Conclusion:**

Dietary protein quantity, quality, and distribution should be considered collectively when looking to optimize protein intake to support muscle strength and function.

## Introduction

Protein is ubiquitous in the human diet, and its adequate supply is essential for maintenance of human health. While it can be used as a source of energy, its primary role is to supply the essential amino acids (EAAs) required to make body proteins (e.g., skeletal and smooth muscle) and the proteins needed for several critical body functions (e.g., enzymes, hormones, immune proteins, etc.). Over the past 2 decades, research investigating the role of protein in the diet has evolved beyond a focus on quantity to also examine the impact or interaction of quality and timing or distribution (1).

In terms of quantity, the optimum amount of dietary protein to support health remains a topic of debate. In the US, 0.8 g of “good-quality” dietary protein per kg body weight per day is the recommended dietary allowance (RDA) for healthy adults (19+ years) (2). It is based on a meta-analysis of nitrogen balance studies (3) and is meant to serve as the minimum requirement to achieve a neutral nitrogen balance for 97.5% of the adult population. It is important to note that it is not based on data to support a specific health outcome or physiological benefit, and some have shown it to be an underestimate of the true requirement using more recent methodologies (4). As such, research has evolved to focus on a variety of metabolic and health-related outcomes, with a growing body of science supporting the benefits of higher dietary protein intakes in adults primarily related to supporting muscle anabolism (muscle protein synthesis and positive net protein balance) and maintenance of lean body mass, muscle strength, and physical function (5–9). Given the decline in these outcomes that occur with aging, a focus of this work has been on the role of dietary protein to mitigate the development of sarcopenia (the deterioration of skeletal muscle mass and strength/physical function) in older adults. Two separate international expert groups have recommended dietary protein intakes in the range of 1.0–1.2 g/kg/day as a minimum dietary protein intake for healthy older adults, 1.2–1.5 g/kg/day for older adults with acute or chronic illness and up to 2.0 g/kg for those with severe illness or injury or marked malnutrition (10, 11).

The quality of dietary protein, defined as its ability to meet the body’s metabolic demand for amino acids and nitrogen (12), is another important factor to consider. Dietary protein quality is based on 3 factors: amino acid composition, the digestibility of the dietary protein, and the bioavailability of the digested and absorbed amino acids derived from that dietary protein. Dietary protein from animal sources (e.g., dairy, eggs, and meat) and from soy are generally of higher quality than most plant-based dietary proteins (e.g., wheat, rice, and pea) (13). A recent systematic review on the impact of dietary protein source and quality for skeletal muscle anabolism (5) found a benefit of higher-quality dietary protein for stimulating muscle protein synthesis at rest and following resistance exercise in both young and older adults. Higher-quality dietary protein was also associated with greater gains in strength accompanying routine resistance exercise training; however, there was no effect on changes in lean body mass. One of the limitations of the available literature on this topic is that a majority of these studies have been conducted comparing select isolated dietary protein foods or ingredients (e.g., milk, eggs, beef, whey, casein, and soy), which are all generally high quality. Additional research is needed to examine a broader range of dietary protein quality in the context of mixed dietary patterns primarily made up of whole foods.

The distribution of dietary protein across meals and snacks is another consideration that has been proposed to impact dietary protein metabolism and subsequent changes in muscle mass, strength, and function (14–16). A dietary plan that includes 25–30 g of high-quality dietary protein per meal has been recommended by some research groups (14, 15). However, the data to date on it providing an advantage over a more skewed dietary protein intake pattern has been mixed (6, 17).

Skeletal muscle is required for performance of activities of daily living, and optimal muscle strength and function are important elements for health and physical performance (18–20). Aging is associated with a progressive loss of skeletal muscle mass (21, 22) and strength, and an adequate amount of dietary protein/essential amino acids is critical for muscle protein synthesis to preserve muscle mass and therefore may limit the risk of age-related disability. Higher dietary protein intake has been shown to be associated with improved physical performance (8, 23) and muscle strength, and EAA, particularly leucine, may play a critical role in augmenting protein synthesis (24–27), and in modulating functional outcomes (28–30).

A recent systematic review from Lunt et al. (31), who examined the clinical usefulness of muscle mass and strength measures in older people, found that hand grip strength was the most studied measure and was associated with mobility, balance, and activities of daily living outcomes. However, only a handful of large population-based cross-sectional studies have examined the association between dietary protein and hand grip strength, and results have been inconsistent (32–41). Additionally, most of these studies have primarily focused on middle aged and older adults and have not investigated the effect of dietary protein from different sources. The objective of this study was to determine the association of dietary protein intake with hand grip strength and to analyze the effect of dietary protein type and intake patterns in young and older adults using a large nationally representative national database of the American population. We hypothesized that dietary protein intake would be positively associated with hand grip strength, and this association would be modified by dietary protein amount, dietary protein type/quality (total, animal, and plant), and its distribution across meals.

## Materials and Methods

We used dietary intake and grip strength data from adults aged 19 + years participating in the National Health and Nutrition Examination Survey (NHANES) 2011–2012 and 2013–2014 cycles (only cycles with grip strength data) with exclusions for pregnant and lactating women, and those with incomplete dietary recall or missing grip strength data (Supplementary Figure 1). The NHANES survey uses a complex, multistage probability design to produce a nationally representative sample of non-institutionalized civilian population in U.S. The NHANES 2011–2012 and 2013–2014 cycles included oversampling of subgroups, including some racial/ethnic minorities: (Hispanic, non-Hispanic black, and non-Hispanic Asian); non-Hispanic white at or below 130% of poverty level; and those aged 80 years and older. The National Center for Health Statistics Research Ethics Review Board approved the protocol, and all participants or proxies provided written informed consent. The overall survey examination response rate was ∼70%, and additional details of NHANES are available elsewhere (42). This study was a secondary data analysis of publicly available federal data without personal identifiers, therefore, did not require Institutional Review Board review.

Dietary intake data with reliable in person 24-h recall dietary interviews administered using the United States Department of Agriculture (USDA)’s automated multiple-pass method were used to estimate intake (43). While two dietary recalls were collected, the first day dietary recall was collected with validated methods; therefore, only this dietary recall was used in all analyses. The NHANES cycle-specific USDA Nutrient Database for Standard Reference in conjunction with the respective Food and Nutrient Database for Dietary Studies and Food Patterns Equivalent Database (44, 45) were used to determine intake of dietary protein (g); dietary protein type: total protein (TP), animal protein including dairy protein (AP) and plant protein (PP); and leucine (g) from foods consumed by the NHANES participants. Dietary protein intake per eating occasion (breakfast, lunch, dinner, and snack) were determined by grouping daily dietary protein intake into (1) breakfast; (2) lunch/brunch; (3) dinner/supper; and (4) snack from a list of different types of self-defined eating occasions provided to the NHANES participants for selection.

Grip strength was measured using a digital handheld Takei dynamometer (Model T.K.K.5401). Following a practice trial, the participants were asked to squeeze the dynamometer as hard as possible using one hand and exhaling while squeezing to avoid build-up of intra-thoracic pressure. Grip strength was measured 3 times in each hand in a standing position. The combined grip strength (in kg) was calculated as the sum of the highest reading from each hand (42). This variable was not calculated for participants who only performed the test on one hand.

All analyses were performed using SAS 9.4 (SAS Institute, Cary, NC, United States) and SUDAN 11 (RTI International, Research Triangle Park, NC, United States). The data were adjusted for complex sample design of NHANES, using appropriate survey weights, strata, and primary sampling units and examination weights to ensure nationally representative estimates following the NHANES analytical guidelines.^1^ Subjects were grouped (1) by the dietary protein (different types: TP, AP, and PP) and leucine intake quartiles; (2) below and above 20 g dietary protein at different meal occasions; and (3) by the number of meal occasions with over 20 g dietary protein. Least square mean (LSM) and standard error (SE) were generated *via* linear regression analyses for grip strength within quartiles of intake, and for adults below and above 20 g dietary protein intake at each meal occasion, and for the number of meal occasions with over 20 g of dietary protein. Linear regression analyses were used to assess the association of dietary protein and leucine intake quartiles with combined grip strength; to assess whether consuming ≥ 20 g of dietary protein at meals and snacks, or at one or more meals was related to combined grip strength (the r-square of models in those 19 + y was typically around 65%). Based on Dietary Reference Intake groups, separate analyses were conducted for adults (gender combined) aged 19 + years (*N* = 9,214), 19–50 years (*N* = 5,091) and 51 + years (*N* = 4,123), after adjusting data for age, gender, and ethnicity (Hispanic, White, Black, Asian, and other) and for 19 + years men and women after adjusting for age and ethnicity. Physical activity was also added to the above covariate set as an additional sensitivity analysis. Significance for all statistical evaluations was set at *p* < 0.05.

## Results

Table 1 shows the mean quartile of dietary protein and leucine intake in adults by age groups. Mean intake of TP among adults aged 19 + years was 83.6 ± 0.5 g/day, and 2/3rd of this was from animal sources (including dairy). Adults aged 19–50 years consumed about 12 g more TP than older adults aged 51 + years. Men aged 19 + years consumed more TP than women aged 19 + years (98.8 ± 0.7 vs. 68.5 ± 0.5 g/day total protein).

**TABLE 1:** Overall mean and mean intake of quartiles of intake (g) of different dietary protein sources and leucine among gender combined adults by age groups, NHANES 2011–2014.

	Mean intake	Quartile 1	Quartile 2	Quartile 3	Quartile 4
**Dietary total protein (TP)**					
19 + years of age	83.6 ± 0.5	41.5 ± 0.6	64.8 ± 0.5	88.8 ± 0.7	128 ± 1
19–50 years of age	89.0 ± 0.8	42.4 ± 0.6	68.2 ± 0.9	94.7 ± 1.3	139 ± 2
51 + years of age	76.5 ± 0.7	40.1 ± 0.9	61.3 ± 0.7	81.9 ± 0.9	115 ± 1
**Dietary animal protein (AP)**					
19 + years of age	56.1 ± 0.5	21.4 ± 0.4	40.4 ± 0.4	59.5 ± 0.7	93.2 ± 1.2
19–50 years of age	60.8 ± 0.8	22.4 ± 0.6	43.3 ± 0.8	65.2 ± 1.0	100 ± 2
51 + years of age	50.1 ± 0.7	20.1 ± 0.7	37.5 ± 0.7	53.5 ± 0.9	82.8 ± 1.6
**Dietary plant protein (PP)**					
19 + years of age	27.4 ± 0.2	12.4 ± 0.2	20.5 ± 0.2	28.8 ± 0.3	43.7 ± 0.5
19–50 years of age	28.2 ± 0.3	12.5 ± 0.3	20.7 ± 0.3	29.6 ± 0.4	45.3 ± 0.6
51 + years of age	26.4 ± 0.4	12.3 ± 0.2	20.3 ± 0.4	28.1 ± 0.4	41.9 ± 0.7
**Leucine**					
19 + years of age	6.55 ± 0.04	3.17 ± 0.05	5.03 ± 0.05	6.93 ± 0.07	10.2 ± 0.1
19–50 years of age	7.03 ± 0.07	3.30 ± 0.06	5.35 ± 0.07	7.46 ± 0.08	11.1 ± 0.1
51 + years of age	5.94 ± 0.06	3.04 ± 0.08	4.73 ± 0.08	6.37 ± 0.07	9.18 ± 0.15

*Dietary animal protein includes dairy protein. N = 9,954 for age 19 + years, 5,432 for age 19–50 years and 4,522 for age 51 + years.*

Table 2 shows the data for combined grip strength with increasing quartiles of different dietary protein types (TP, AP, and PP) in gender combined adults by age groups. For all adults aged 19 + years, combined grip strength increased with increasing quartiles of TP, AP, and PP. However, the increase in combined grip strength was more evident for increasing quartiles of AP than for PP, and the significant increases were detected at quartile 2 for AP and at quartile 3 for TP and PP. Similar increases in grip strength with increasing quartiles of protein were obtained when data were separately analyzed for adults aged 19–50 years for TP, AP, and PP, and for adults aged 51 + years for TP and AP. However, for adults aged 51 + years, increase in combined grip strength with increasing quartiles of PP did not reach statistical significance. Overall, adding physical activity as a covariate had virtually no impact on conclusions drawn from results of this sensitivity analysis (data not shown). Similar associations between dietary protein type and grip strength were also observed when analyzed for men and women separately, except that the association of PP did not reach statistical significance for men aged 51 + years and for women aged 19–50 and 51 + years (Tables 3, 4), and adding physical activity as a covariate had virtually no impact on conclusions drawn from results, except for the association of PP in women aged 19 + years became insignificant (data not shown).

**TABLE 2:** Association of dietary protein intake with combined grip strength (kg) in gender combined adults by age groups, NHANES 2011–2014.

	Dietary protein intake quartiles				Quartile trend	
		
	Quartile 1	Quartile 2	Quartile 3	Quartile 4	β	*p*
**19 + years of age**						
Total protein	71.3 ± 0.4	72.6 ± 0.5	73.3 ± 0.4*	75.6 ± 0.5*	1.34 ± 0.19	<0.0001
Animal protein	71.3 ± 0.4	72.7 ± 0.3*	73.4 ± 0.5*	75.3 ± 0.5*	1.27 ± 0.19	<0.0001
Plant protein	72.2 ± 0.3	72.5 ± 0.4	73.7 ± 0.3*	74.3 ± 0.7*	0.76 ± 0.20	0.0007
**19–50 years of age**						
Total protein	77.9 ± 0.5	78.6 ± 0.5	79.1 ± 0.7	81.5 ± 0.6*	1.14 ± 0.27	0.0002
Animal protein	77.6 ± 0.4	79.5 ± 0.5*	78.6 ± 0.6	81.4 ± 0.6*	1.06 ± 0.25	0.0002
Plant protein	78.4 ± 0.4	78.4 ± 0.5	79.7 ± 0.4^#^	80.6 ± 0.8^#^	0.77 ± 0.30	0.0166
**51 + years of age**						
Total protein	63.7 ± 0.7	64.8 ± 0.7	65.6 ± 0.6^#^	66.6 ± 0.5*	0.95 ± 0.26	0.0008
Animal protein	63.3 ± 0.6	65.3 ± 0.6*	65.1 ± 0.7^#^	67.0 ± 0.5*	1.08 ± 0.27	0.0003
Plant protein	64.4 ± 0.5	65.2 ± 0.6	65.9 ± 0.7^#^	65.3 ± 0.6	0.33 ± 0.23	0.1570

Data adjusted for age, gender, and ethnicity; and presented as least square mean ± standard error. Refer to Table 1 for dietary protein intake quartiles. Dietary animal protein includes dairy protein. * and ^#^ indicate significant difference from Quartile 1 at p < 0.01 and p < 0.05. N = 9,214 for age 19 + years, 5,091 for age 19–50 years, and 4,123 for age 51 + years.

**TABLE 3:** Association of dietary protein intake with combined grip strength (kg) in male adults by age groups, NHANES 2011–2014.

	Dietary protein intake quartiles				Quartile trend	
		
	Quartile 1	Quartile 2	Quartile 3	Quartile 4	β	*p*
**19 + years of age**						
Total protein	87.8 ± 0.7	89.1 ± 0.7	90.7 ± 0.9*	93.2 ± 0.8*	1.76 ± 0.32	<0.0001
Animal protein	88.1 ± 0.8	89.7 ± 0.8	90.2 ± 0.8	92.9 ± 0.7*	1.51 ± 0.34	0.0001
Plant protein	88.4 ± 0.8	90.0 ± 0.8	90.5 ± 0.8^#^	92.1 ± 0.8*	1.14 ± 0.28	0.0003
**19–50 years of age**						
Total protein	94.3 ± 0.7	94.9 ± 1.1	96.0 ± 1.0	98.9 ± 0.9*	1.49 ± 0.40	0.0007
Animal protein	94.2 ± 0.8	95.6 ± 1.1	95.8 ± 1.0	98.5 ± 0.8*	1.34 ± 0.40	0.0019
Plant protein	93.7 ± 0.8	95.9 ± 0.9	96.4 ± 0.9^#^	98.1 ± 0.9*	1.34 ± 0.38	0.0013
**51 + years of age**						
Total protein	79.9 ± 1.6	82.1 ± 1.2	81.9 ± 1.1	83.0 ± 0.9^#^	0.91 ± 0.47	0.0609
Animal protein	79.7 ± 1.7	81.0 ± 1.1	83.2 ± 1.2	82.8 ± 0.9	1.13 ± 0.49	0.0262
Plant protein	81.6 ± 1.5	81.1 ± 0.8	81.6 ± 1.3	82.4 ± 0.8	0.29 ± 0.41	0.4819

Data adjusted for age, gender, and ethnicity; and presented as least square mean ± standard error. Refer to Table 1 for protein intake quartiles. Dietary animal protein includes dairy protein. * and ^#^ indicate significant difference from Quartile 1 at p < 0.01 and p < 0.05. N = 4,631 for age 19 + years, 2,606 for age 19–50 years, and 2,025 for age 51 + years.

**TABLE 4:** Association of dietary protein intake with combined grip strength (kg) in female adults by age groups, NHANES 2011–2014.

	Dietary protein intake quartiles				Quartile trend	
		
	Quartile 1	Quartile 2	Quartile 3	Quartile 4	β	*p*
**19 + years of age**						
Total protein	54.5 ± 0.4	55.9 ± 0.4^#^	57.0 ± 0.4*	57.0 ± 0.4*	0.88 ± 0.20	0.0001
Animal protein	54.6 ± 0.4	56.5 ± 0.4*	56.3 ± 0.4*	57.0 ± 0.4*	0.72 ± 0.15	0.0001
Plant protein	55.5 ± 0.4	55.7 ± 0.3	56.3 ± 0.4	56.8 ± 0.5	0.45 ± 0.22	0.0478
**19–50 years of age**						
Total protein	59.4 ± 0.6	61.1 ± 0.4^#^	61.0 ± 0.5	61.4 ± 0.5*	0.57 ± 0.24	0.0237
Animal protein	59.6 ± 0.5	60.8 ± 0.5	61.4 ± 0.5^#^	61.2 ± 0.5^#^	0.55 ± 0.23	0.0233
Plant protein	60.5 ± 0.5	60.4 ± 0.6	60.9 ± 0.6	61.1 ± 0.7	0.23 ± 0.28	0.4193
**51 + years of age**						
Total protein	49.0 ± 0.5	50.6 ± 0.7	51.2 ± 0.5*	51.5 ± 0.5*	0.82 ± 0.25	0.0023
Animal protein	49.3 ± 0.5	50.9 ± 0.7	51.0 ± 0.4^#^	51.3 ± 0.5*	0.62 ± 0.19	0.0024
Plant protein	49.6 ± 0.6	50.8 ± 0.5	51.1 ± 0.6^#^	51.0 ± 0.5	0.45 ± 0.26	0.0911

Data adjusted for age, gender, and ethnicity; and presented as least square mean ± standard error. Refer to Table 1 for dietary protein intake quartiles. Dietary animal protein includes dairy protein. * and ^#^ indicate significant difference from Quartile 1 at p < 0.01 and p < 0.05. N = 4,583 for age 19 + years, 2,485 for age 19–50 years, and 2,098 for age 51 + years.

Combined grip strength also increased significantly with increasing quartiles of total leucine among gender combined adults aged 19 + years with significant increases detected at quartile 2. In general, significant increases in grip strength with increasing quartiles of total leucine were also observed when the data were analyzed by age and gender groups, except for male adults aged 51 + years where the association did not reach statistical significance (Table 5), and adding physical activity as a covariate had virtually no impact on conclusions drawn from results.

**TABLE 5:** Association of leucine intake with combined grip strength (kg) in adults by age and gender groups, NHANES 2011–2014.

	*N*	Leucine intake quartiles				Quartile trend	
			
		Quartile 1	Quartile 2	Quartile 3	Quartile 4	β	*p*
**Gender combined**							
19 + years of age	9,214	71.2 ± 0.4	72.9 ± 0.5*	73.2 ± 0.5*	75.5 ± 0.5*	1.33 ± 0.23	<0.0001
19–50 years of age	5,091	77.7 ± 0.4	78.9 ± 0.6	78.9 ± 0.7	81.6 ± 0.6*	1.18 ± 0.27	0.0001
51 + years of age	4,123	63.3 ± 0.6	65.4 ± 0.6*	65.4 ± 0.6*	66.8 ± 0.5*	1.05 ± 0.27	0.0004
**Male**							
19 + years of age	4,631	88.0 ± 0.7	89.3 ± 0.8	90.8 ± 0.8^#^	92.8 ± 0.8*	1.59 ± 0.31	< 0.0001
19–50 years of age	2,606	94.0 ± 0.7	95.4 ± 1.0	95.8 ± 1.0	98.9 ± 1.0*	1.50 ± 0.41	0.0009
51 + years of age	2,025	79.5 ± 1.5	82.1 ± 1.0	82.4 ± 1.1	82.7 ± 0.9^#^	0.96 ± 0.48	0.0518
**Female**							
19 + years of age	4583	54.3 ± 0.4	56.3 ± 0.4*	56.8 ± 0.5*	57.0 ± 0.3*	0.86 ± 0.18	0.0001
19–50 years of age	2,485	59.4 ± 0.5	60.7 ± 0.4^#^	61.5 ± 0.5^#^	61.4 ± 0.5*	0.68 ± 0.24	0.0088
51 + years of age	2,098	48.8 ± 0.5	50.6 ± 0.7^#^	51.7 ± 0.5*	51.3 ± 0.5*	0.85 ± 0.23	0.0009

Data adjusted for age, gender, and ethnicity; and presented as least square mean ± standard error. Refer to Table 1 for leucine intake quartiles. * and ^#^ indicate significant difference from Quartile 1 at p < 0.01 and p < 0.05.

We also compared combined grip strength among adults consuming over 20 g dietary protein at one or more meal occasions with adults consuming no meals with over 20 g per meal occasion (Table 6). Combined grip strength increased with increasing the number of meal occasions containing more than 20 g of dietary protein, and significant increases were detected for two meals compared to zero meals for gender combined adults aged 19+, 19–50, and 51+ years. Similar positive associations between the number of meals with over 20 g of dietary protein were also observed when data were analyzed for men and women separately, except for men and women aged 51 + years where the association did not reach statistical significance (Table 6). Adding physical activity as a covariate had virtually no impact on conclusions drawn from results. Combined grip strength was also higher for adults aged 19 + years consuming over 20 g dietary protein at lunch, dinner, or snacks than those consuming less than 20 g dietary protein for those meal occasions. Similarly, adults aged 19–50 years consuming over 20 g dietary protein at lunch, dinner, or snack also had higher combined grip strength than those with below 20 g for those meal occasions. However, for adults aged 51 + years, combined grip strength was only higher for those consuming over 20 g dietary protein at lunch as compared to those with below 20 g protein at lunch (Supplementary Table 1). However, results for dinner and snacks were no longer significant when physical activity was added as additional covariate in a sensitivity analysis. When data were analyzed for men and women separately, combined grip strength was only higher for men aged 19 + years consuming over 20 g dietary protein at lunch and for women aged 19 + years consuming over 20 g dietary protein at dinner or snacks compared to those consuming less than 20 g dietary protein at the respective meals (Supplementary Table 2).

**TABLE 6:** Association of combined grip strength (kg) with the number of meals with above 20 g dietary protein intakes among adults aged 19 + years by gender groups, NHANES 2011–2014.

	Zero meals		One meal		Two meals		Three + meals		Group trend	
					
	*N*	Grip strength	*N*	Grip strength	*N*	Grip strength	*N*	Grip strength	β	*p*
**Gender combined**										
19 + years of age	909	71.6 ± 0.4	3,487	72.1 ± 0.4	3,680	73.8 ± 0.4*	1,138	75.7 ± 0.6*	1.50 ± 0.20	<0.0001
19–50 years of age	398	77.5 ± 0.6	1,766	78.5 ± 0.4	2,173	79.2 ± 0.5^#^	754	82.0 ± 0.7*	1.41 ± 0.25	<0.0001
51 + years of age	511	64.2 ± 0.6	1,721	64.4 ± 0.7	1,507	66.2 ± 0.5^#^	384	65.8 ± 1.1	0.91 ± 0.37	0.0202
**Male**										
19 + years of age	222	88.9 ± 1.5	1,483	88.0 ± 0.7	2,126	90.5 ± 0.5	800	93.2 ± 1.0^#^	2.18 ± 0.45	<0.0001
19–50 years of age	91	95.2 ± 1.8	741	94.0 ± 0.7	1,251	95.6 ± 0.7	523	99.5 ± 1.0	2.22 ± 0.55	0.0003
51 + years of age	131	82.0 ± 2.0	742	80.1 ± 1.4	875	82.5 ± 0.8	277	82.7 ± 1.4	1.06 ± 0.77	0.1758
**Female**										
19 + years of age	687	54.7 ± 0.6	2,004	55.8 ± 0.3	1,554	57.0 ± 0.4*	338	56.7 ± 0.7^#^	0.86 ± 0.26	0.0026
19–50 years of age	307	59.0 ± 0.6	1,025	60.9 ± 0.3*	922	61.1 ± 0.4*	231	61.3 ± 0.9^#^	0.65 ± 0.27	0.0248
51 + years of age	380	49.6 ± 0.7	979	50.3 ± 0.4	632	51.8 ± 0.5^#^	107	49.5 ± 1.3	0.69 ± 0.36	0.0633

Data adjusted for age, gender, and ethnicity; and presented as least square mean ± standard error. * and ^#^ indicate significant difference from Zero Meals at p < 0.01 and p < 0.05.

## Discussion

The current cross-sectional analysis of data from the NHANES 2011–2014 demonstrated a significant positive association between both total dietary protein intake and dietary protein type and grip strength among US adults by age and gender groups. Additionally, grip strength was associated with the number of meals with more than 20 g dietary protein per meal and meal occasion.

Dietary protein intake in amounts moderately higher than the RDA have been reported to support maintenance of muscle mass and strength, and physical functioning in adults/older adults in a number of observational studies (36, 41, 46–48). However, data from clinical trials are equivocal and often reported no benefits of protein supplementation (49, 50). In controlled feeding studies of 3-month duration, dietary protein intake at or below the RDA resulted in loss of lean muscle mass in older adults (51, 52). Inconsistent improvements in muscle mass and physical function with dietary protein intakes above RDA were reported in other studies with older adults (9, 53–55). Heterogeneity in study design, including health status of subjects (e.g., healthy vs. frail, with or without impairments in mobility/physical function) and background habitual protein intake, inclusion of resistance exercise training, study duration, etc., could be responsible for the inconsistencies in these studies.

There is an increased focus on muscle strength and physical function as these have become the primary indicators of sarcopenia outlined in the latest the European consensus guidelines (56). Additionally, physical function was shown to be more related to health and mortality than pure muscle mass in a recent cross-sectional study (18), and a recent systematic review on the clinical usefulness of muscle mass and strength measures in older people found that grip strength was the most studied measure and was associated with mobility, balance, and activities of daily living outcomes (31). Grip strength has also been shown to be inversely associated with all-cause mortality in the UK Biobank prospective cohort study (57). We found that increasing intake of dietary protein was associated with significantly higher grip strength in our analysis of NHANES. Similar positive associations between dietary protein intake and grip strength were also reported in earlier cross-sectional studies with NHANES and other databases (38–41).

Dietary protein quality, which can be equated to the efficiency with which a food can deliver the full array of EAAs in sufficient amounts that are highly digestible and bioavailable in support of the body’s EAA requirements, has been shown to influence muscle protein synthesis (5). However, there is limited and inconsistent data on the impact of dietary protein quality on muscle strength. A meta-analysis of 16 clinical trials concluded that while dietary animal protein tends to have a more favorable effect on lean mass compared to dietary plant protein, dietary protein source was not likely to have an impact on muscle strength (7). Higher dietary protein intake regardless of quality was associated with less decrease or gain in hand grip strength. However, when the analysis was stratified by age, AP, but not PP was significantly linked to grip strength in adults aged 51 + years, whereas the relationship of either animal or plant dietary protein was not significant in adults aged less than 60 years in an analysis of Framingham Offspring cohort (38). In an earlier analysis of NHANES data, dietary plant protein was positively associated with hand grip strength. This association was attenuated after adjustments for fruit/vegetable intake (36). Increasing quartiles of both animal and plant dietary proteins were associated with increased hand grip strength in all adults, but the increase was significant at a lower quartile for AP than for PP (quartile 2 AP vs. quartile 3 for PP) in the present study. Additionally, in adults over 51 years of age, the association with grip strength was observed only for AP but not for PP. These results suggest that dietary protein quality may be more important for older vs. younger adults. This can be due to a combination of anabolic resistance in older adults, which results in a higher EAA and leucine threshold to stimulate and maximize muscle protein synthesis, higher total dietary protein needs, and lower energy intake/needs, which leads to a heightened importance on the efficiency with which a food/diet can deliver metabolically available EAAs (58, 59). Indeed, a recent modeling study illustrated that dietary patterns, which solely rely on plant-based protein foods, require higher total energy intake to meet EAA requirements in older women compared to those that also incorporate animal-based protein foods (60). This is a result of the lower EAA density (EAA/100 kcal) of most plant-based foods. It should be noted that this study did not take digestibility and bioavailability into account which likely would have further illustrated the impact of differences in protein quality on meal/diet planning. Dietary protein quality also becomes more important in populations with lower total dietary protein intakes. This was illustrated in a recent paper that evaluated the adequacy of population-based dietary protein intakes when corrected for dietary protein quality (61). When analyzed on a gross dietary protein basis, the average daily dietary protein intakes for the majority of the 103 countries examined (primarily in Africa and Asia with an average dietary protein intake < 70 g/day) exceeded the current requirement of 50 g/day (based on the recommended protein intake of 0.8 g/kg/day for 62-kg adult). However, when TP intake was corrected for dietary protein quality, all the 103 country’s average daily protein intakes fell below the requirement.

Leucine, one of the three branched-chain amino acids, has been suggested to be the most potent EAA responsible for modulating postprandial muscle protein synthesis (24, 25) and 2.5–3.0 g leucine per meal has been recommended to help maximize the muscle protein synthesis response (11, 13, 28). Based on the indicator amino acid oxidation method, leucine requirements of healthy older adults were recently suggested to be more than double the amount current national (estimated average requirement of 34 mg/kg/day) and international (39 mg/kg/day) recommendations (62). The other EAAs are also needed to support the ongoing stimulation of muscle protein synthesis (MPS) and subsequent long-term changes in muscle mass. That said, the value of dietary leucine or supplementary leucine appears to be more relevant in cases where protein (single meal and/or total diet) is suboptimal. For example, lower amounts of TP with high leucine content can stimulate MPS to a similar extent as a larger amount of protein that provides a similar amount of leucine (25, 63). Leucine may also be used as a proxy for protein quality of a meal or diet. Indeed, the results presented here showed that grip strength was positively linked with increasing quartiles of leucine intake, which could represent a proxy for protein quantity and quality.

Dietary protein distribution across meals related to a threshold level of dietary protein per meal to maximize muscle protein synthesis, and help to support muscle mass, strength, and function has been a recent area of focus in an ongoing effort to optimize dietary protein recommendations, particularly in relation to their ability to reduce the risk of sarcopenia. The rationale for per-meal recommendations is based on the saturable dose–response relationship that has been established between dietary protein feeding and the muscle protein synthesis response, whereby increased amounts of dietary protein beyond this dose do not further increase muscle protein synthesis, and these excess amino acids are primarily oxidized (15, 17). Paddon-Jones and Rasmussen were one of the first to propose an approach of consuming 25–30 g of high-quality dietary protein per meal to reduce the risk of sarcopenia (14). This recommendation was later supported in a position paper from the PROT-AGE Study Group (11). However, recent reviews have concluded the data to date are equivocal (6, 15, 17), with more work needed to better address considerations regarding the per-meal dietary protein dose (was a dose demonstrated to maximize muscle protein synthesis used), how the dietary protein is delivered (as part of a mixed meal vs. single food/protein ingredient), total dietary protein intake (per-meal dietary protein strategies are likely more important at lower total dietary protein intakes), and state of energy balance.

Related to our work, an earlier analysis of NHANES 1999–2002 found that knee extension strength was significantly associated with higher frequency of meals with at least 30 g dietary protein (64). In contrast, a more recent analysis of NHANES 2011–2014 reported that consumption of > 20 g of dietary protein at 2 or more meals was not associated with grip strength among a small number of older adults (40). In the present analysis, grip strength was associated with the number of meals with over 20 g of dietary protein. This finding supports those from previous studies, which have shown that the postprandial muscle protein synthesis response was dose-dependent up to 20 g of high-quality, animal-based dietary protein in healthy young individuals (65, 66), whereas > 35-g high-quality, animal-based dietary protein was required maximal stimulation of postprandial muscle protein synthesis in older individuals (67–69). Moore et al. also reported that a lower amount of dietary protein per meal was needed to saturate muscle protein synthesis in young adults (∼0.24 g/kg/meal) than in older adults (0.4 g/kg/meal) (59). As our population spanned younger through older adults, we opted to go with the lower amount to capture what would be deemed as optimal to support maximal MPS *via* provision of high-quality dietary protein. Interestingly, our results also show lunch to be a particularly important meal to achieve 20 g of dietary protein as it relates to grip strength. One possible explanation could be that for a significant amount of the population, lunch was the first meal where more optimal dietary protein targets (total protein, animal protein, and leucine) were hit, whereas breakfast for many was not sufficient to hit these targets and support muscle anabolism. Future studies should more closely examine the impact of consuming > 20 g of dietary protein at lunch vs. other meals on muscle function and strength.

Given the results from this and other studies and the clear connectedness or interdependence of dietary protein quantity, quality, and distribution per meal, we propose to frame assessment of the literature and practical recommendations for optimizing protein intakes in a hierarchical pyramid approach. Quantity forms the base of the pyramid and is the biggest driver of outcomes, whereas quality is second and distribution is third. Accordingly, with higher total dietary protein intake, the quality and distribution will have a lesser impact; however, at lower total energy and dietary protein intakes, quality and then distribution can become more important. This potential hierarchy also suggests that for specific populations (i.e., older adults and adolescent women) and/or specific diets (i.e., those lower in high-quality dietary proteins), dietary protein quality and distribution become more important in dietary guidance and meal planning.

The strengths of this study include the use of a large nationally representative sample and the use of numerous covariates to adjust data to attempt to limit potential confounding. Another strength of the present study is that we examined the link between protein and grip strength across the full adult age span. That said, there is need for more data on middle-aged adults specifically to better delineate when these declines in muscle mass/strength begin and when diet becomes more important. A third strength was that we examined whole foods and actual dietary intakes to address the question of the importance of protein quality, whereas protein supplementation trials primarily use comparisons of single protein sources. Additionally, a variety of questions/variables related to protein intake (quantity, quality, and leucine, and distribution) are addressed in single analysis. A major limitation of this study is the use of a cross-sectional study design, which cannot be used to determine cause and effect. The dietary intake data were self-reported single-day recalls relying on memory and are potentially subject to reporting bias. Amino acid database in NHANES is not complete, and assumptions were needed to expand older data to new intake data. Grip strength is only one measure of strength/function available in NHANES. Ideally, using multiple measures of strength/function would provide a more complete picture. A relatively older dataset NHANES 2011–2014 was used in this study because grip strength measurements were available only in these. There is a clear need for the inclusion of assessments of muscle strength/function in future NHANES datasets to better allow for the assessment of association between protein/diet quality and physical/muscle function as muscle/physical function has become the primary indicator for sarcopenia and is more related to health and mortality than purely muscle mass. Finally, while we accounted for a number of covariates in our statistical models, residual confounding cannot be ruled out. Future analyses should also consider adjustments for intake of other dietary components (e.g., fruits, vegetables, and protein supplements). More clinical trials are needed to help test the relationships identified in this and other observational work in order to sufficiently address the ongoing questions regarding optimizing dietary protein intakes to support health, particularly physical function, in adults.

## Data Availability Statement

Publicly available datasets were analyzed in this study. This data can be found here: https://wwwn.cdc.gov/nchs/nhanes/.

## Author Contributions

MP and CC participated in formulating the research question, design of analyses, interpretation of the data, drafting the manuscript, revising the manuscript, and the approval of the final version. SA participated in interpretation of the data, drafting the manuscript, revising the manuscript, and approval of the final version. VF participated in formulating the research question, design of analyses, dietary data analysis, interpretation of the data, drafting the manuscript, revising the manuscript, and approval of the final version. All authors contributed to the article and approved the submitted version.

## Conflict of Interest

MP and CC are employees of the National Dairy Council, Rosemont, IL, United States. SA as a Principal of NutriScience LLC performs nutrition science consulting for various food and beverage companies and related entities. VF was the Senior Vice President of Nutrition Impact LLC, provides database analyses and food/nutrition consulting to the food/beverage industry, and received a research grant from National Dairy Council to conduct these analyses.

## Publisher’s Note

All claims expressed in this article are solely those of the authors and do not necessarily represent those of their affiliated organizations, or those of the publisher, the editors and the reviewers. Any product that may be evaluated in this article, or claim that may be made by its manufacturer, is not guaranteed or endorsed by the publisher.

## Footnotes

1
https://wwwn.cdc.gov/nchs/data/nhanes/analyticguidelines/11-16-analytic-guidelines.pdf
