# Supplementary material for: Association of Dietary Protein Intake and Grip Strength Among Adults Aged 19+ Years: NHANES 2011–2014 Analysis
Source: Front Nutr. 2022 May 13;9:873512. doi: 10.3389/fnut.2022.873512 (PMC9136219; doi:10.3389/fnut.2022.873512)
Supplement: Supplementary file 1 [file Data_Sheet_1.docx]

Association of dietary protein intake and grip strength among adults aged 19+ years: NHANES 2011-2014 analysis

Supplementary Material

**Supplemental Table 1**. Comparison of combined grip strength (kg) between below and above 20 g dietary protein intakes at different meals among gender combined adults by age groups, NHANES 2011-2014.

|  | 20 g or less of Total Dietary Protein | | Over 20 g of Total Dietary Protein | | Difference | P |
| --- | --- | --- | --- | --- | --- | --- |
|  | N | Grip Strength | N | Grip Strength |  |  |
| 19+ years of age |  |  |  |  |  |  |
| Breakfast | 6,908 | 73.0 ± 0.3 | 2,306 | 73.9 ± 0.5 | 0.87 ± 0.50 | 0.0940 |
| Lunch | 4,957 | 72.2 ± 0.3 | 4,257 | 74.3 ± 0.4 | 2.07 ± 0.40 | <0.0001 |
| Dinner | 2,888 | 72.4 ± 0.4 | 6,326 | 73.5 ± 0.3 | 1.11 ± 0.49 | 0.0315 |
| Snack | 7,723 | 73.0 ± 0.3 | 1,491 | 74.3 ± 0.6 | 1.33 ± 0.57 | 0.0264 |
| 19-50 years of age |  |  |  |  |  |  |
| Breakfast | 3,776 | 79.1 ± 0.4 | 1,315 | 79.9 ± 0.6 | 0.82 ± 0.75 | 0.2818 |
| Lunch | 2,522 | 78.3 ± 0.3 | 2,569 | 80.2 ± 0.5 | 1.83 ± 0.53 | 0.0016 |
| Dinner | 1,456 | 78.4 ± 0.4 | 3,635 | 79.6 ± 0.4 | 1.26 ± 0.54 | 0.0271 |
| Snack | 4,149 | 79.0 ± 0.3 | 942 | 80.5 ± 0.6 | 1.47 ± 0.64 | 0.0291 |
| 51+ years of age |  |  |  |  |  |  |
| Breakfast | 3,132 | 65.1 ± 0.4 | 991 | 65.6 ± 0.8 | 0.46 ± 0.76 | 0.5439 |
| Lunch | 2,435 | 64.5 ± 0.5 | 1,688 | 66.1 ± 0.5 | 1.63 ± 0.52 | 0.0035 |
| Dinner | 1,432 | 64.9 ± 0.7 | 2,691 | 65.4 ± 0.5 | 0.51 ± 0.78 | 0.5238 |
| Snack | 3,574 | 65.2 ± 0.4 | 549 | 65.3 ± 0.9 | 0.07 ± 0.91 | 0.9371 |

Data adjusted for age, gender and ethnicity; and presented as least square mean ± standard error.

**Supplemental Table 2.** Comparison of combined grip strength (kg) between below and above 20 g dietary protein intakes at different meals among adults age 19+ years by gender groups, NHANES 2011-2014.

|  | 20 g or less of Dietary Total Protein | Over 20 g of Total Dietary Protein | Difference | P |
| --- | --- | --- | --- | --- |
| Male |  |  |  |  |
| Breakfast | 89.8 ± 0.5 | 91.3 ± 0.7 | 1.55 ± 0.79 | 0.0578 |
| Lunch | 88.3 ± 0.6 | 91.7 ± 0.6 | 3.36 ± 0.73 | 0.0001 |
| Dinner | 89.7 ± 0.8 | 90.4 ± 0.5 | 0.64 ± 0.79 | 0.4241 |
| Snack | 90.0 ± 0.5 | 91.3 ± 0.9 | 1.39 ± 0.97 | 0.1604 |
| Female |  |  |  |  |
| Breakfast | 56.1 ± 0.2 | 56.1 ± 0.5 | 0.00 ± 0.49 | 0.9924 |
| Lunch | 55.9 ± 0.3 | 56.4 ± 0.3 | 0.59 ± 0.39 | 0.1463 |
| Dinner | 55.2 ± 0.4 | 56.6 ± 0.3 | 1.44 ± 0.48 | 0.0057 |
| Snack | 55.9 ± 0.2 | 57.1 ± 0.5 | 1.15 ± 0.53 | 0.0373 |

Data adjusted for age, gender and ethnicity; and presented as least square mean ± standard error.

**Supplemental Figure 1.** Participant flow chart.

NHANES 2011 - 2014

All Subjects 0+ years

n=19,931

Exclusion criteria:

- Age <19 years, n=8,290
- Incomplete dietary recall, n=1,517
- Pregnant or lactating female, n=170
- Missing grip strength data, n=740

NHANES 2011 - 2014

Non-pregnant/not lactating, and with complete dietary recall and grip strength data 19+ years

n=9,214
